# Ultrastructure of mature spermatozoa of three Bucephalidae (*Prosorhynchus longisaccatus, Rhipidocotyle khalili* and *Bucephalus margaritae*) and phylogenetic implications

**DOI:** 10.1051/parasite/2018065

**Published:** 2018-12-07

**Authors:** Papa Ibnou Ndiaye, Bernard Marchand, Cheikh Tidiane Bâ, Jean-Lou Justine, Rodney A. Bray, Yann Quilichini

**Affiliations:** 1 Laboratory of Evolutionary Biology, Ecology and Management of Ecosystems, Faculty of Sciences and Techniques, Cheikh Anta Diop University of Dakar BP 5055 Dakar Senegal; 2 UMR 6134 SPE, CNRS – Università di Corsica, Campus Grimaldi 20250 Corte, Corsica France; 3 Institut Systématique, Évolution, Biodiversité (ISYEB), Muséum national d’Histoire Naturelle, CNRS, Sorbonne Université, EPHE CP 51 57 rue Cuvier 75005 Paris France; 4 Department of Life Sciences, Natural History Museum Cromwell Road London SW7 5BD United Kingdom

**Keywords:** Bucephalidae, Digenea, Phylogeny, Spermatozoon, Ultrastructure

## Abstract

We describe here the mature spermatozoa of three species of bucephalids, namely *Bucephalus margaritae, Rhipidocotyle khalili* and *Prosorhynchus longisaccatus*. This study provides the first ultrastructural data on the genera *Bucephalus* and *Rhipidocotyle* and enabled us to confirm the model of the mature spermatozoon in the Bucephalinae. The spermatozoon exhibits two axonemes with the 9 + “1” pattern of the Trepaxonemata, one of which is very short, lateral expansion, external ornamentation of the plasma membrane located in the anterior extremity of the spermatozoon and associated with cortical microtubules, spine-like bodies, a mitochondrion, and a nucleus. The maximum number of cortical microtubules is located in the anterior part of the spermatozoon. However, more studies are needed to elucidate if spine-like bodies are present in all the Bucephalinae or not. In the Prosorhynchinae, the mature spermatozoon exhibits a similar ultrastructural pattern. Some differences are observed, particularly the axoneme lengths and the arrangement of the spine-like bodies. The posterior extremity of the spermatozoon in the Bucephalinae exhibits only the nucleus, but prosorhynchines have microtubules.

## Abstract (fr)

Dans cette étude, nous décrivons les spermatozoïdes mûrs de trois espèces de bucephalidés, à savoir *Bucephalus margaritae, Rhipidocotyle khalili* et *Prosorhynchus longisaccatus*. Ces premières données ultrastructurales pour les genres *Bucephalus* et *Rhipidocotyle* nous ont permis de confirmer le modèle du spermatozoïde chez les Bucephalinae. Le spermatozoïde comprend deux axonèmes de type 9 + “1” des Trepaxonemata, l’un d’entre eux étant très court, une expansion latérale, une ornementation externe de la membrane plasmique située au niveau de l’extrémité antérieure du spermatozoïde et associée avec des microtubules corticaux, des corps en forme d’épines, une mitochondrie et un noyau. Le nombre maximum de microtubules corticaux est situé dans la partie antérieure du spermatozoïde. Toutefois, des études supplémentaires seraient nécessaires pour vérifier si les corps en forme d’épines sont présents chez tous les Bucephalinae. Chez les Prosorhynchinae, le spermatozoïde mûr présente une structure similaire. Quelques différences sont toutefois observées, notamment au niveau de la longueur des axonèmes et de la disposition des corps en forme d’épines. L’extrémité postérieure du spermatozoïde chez les Bucephalidae est caractérisée par la présence du noyau seul, alors que les Prosorhynchinae ont des microtubules.

## Introduction

The Bucephalidae Poche, 1907 (Platyhelminthes: Digenea) is a large cosmopolitan family that is parasitic in marine, brackish-water and freshwater fishes. It currently comprises 25 genera and 380 nominal species. It is now considered to be the single family within the superfamily Bucephaloidea [19, 21]. These parasites have been reported from various fish species from over one hundred families. The systematic placement of the Bucephalidae within the Digenea has been debated since early studies considered the “gasterostomes” to be distinct from most other digenean groups. Later studies on life-cycles (e.g. [11]), and particularly molecular studies (e.g. [20]), have clearly shown that they are not basal, but closely related to other digenean groups, most notably the superfamily Gymnophalloidea. Nevertheless, more morphological and molecular studies are needed to improve our knowledge on the taxonomic and phylogenetic relationships among the bucephalids.

Today, ultrastructural studies of the mature spermatozoon of Platyhelminthes have provided useful characters for the understanding of phylogenetic relationships between Platyhelminthes [1, 9, 12]. In the Bucephalidae, ultrastructural data on the mature spermatozoon exist for four species belonging to three genera, namely the bucephalines *Prosorhynchoides arcuatus* [10], *Prosorhynchoides gracilescens* published as *Bucephaloides gracilescens* [5], *Pseudorhipidocotyle elopichthys* [26] and the prosorhynchine *Prosorhynchus aculeatus* [14]. We agree with Kacem and Miquel [10] that the ultrastructural studies of the spermatozoa of *B. gracilescens* [5] and *P. elopichthys* [26] provide only limited information. In this study, we provide ultrastructural data on the mature spermatozoon in the Bucephalidae with a study of two bucephalines, *Bucephalus margaritae* and *Rhipidocotyle khalili* and one prosorhynchine, *Prosorhynchus longisaccatus.* This is the first ultrastructural study of spermatozoa in the genera *Bucephalus* and *Rhipidocotyle.*

## Materials and methods

### Material collection

Adult digeneans were obtained from the digestive tract of fish off New Caledonia, South Pacific, using the “wash” method [8]. Specimens of *Rhipidocotyle khalili* Nagaty, 1937 were obtained from *Sphyraena putnamae* (Sphyraenidae), caught September 1st, 2009 off Récif Toombo, off Nouméa; specimens from the same fish were described and are deposited in the MNHN collection as JNC3035A [2]. Specimens of *Prosorhynchus longisaccatus* Durio & Manter, 1968 were collected from *Epinephelus maculatus* (Serranidae), caught September 16th, 2009 off Récif Toombo; specimens from the same fish were described and are deposited as MNHN JNC3052 [3]. Specimens of *Bucephalus margaritae* Ozaki & Ishibashi, 1934 were collected from *Carangoides fulvoguttatus* (Carangidae), bought May 20th, 2010 at the fish market in Nouméa; voucher slides from the same fish are deposited as MNHN JNC3167B.

### Transmission electron microscopy

Live worms were rinsed with a 0.9% NaCl solution and fixed in cold (4 °C) 2.5% glutaraldehyde in a 0.1 M sodium cacodylate buffer at pH 7.2, rinsed in 0.1 M sodium cacodylate buffer at pH 7.2, post-fixed in cold (4 °C) 1% osmium tetroxide in the same buffer for 1 h, dehydrated in ethanol and propylene oxide, embedded in Spurr’s resin and polymerized at 60 °C for 24 h.

Ultrathin sections (60–90 nm thick) were obtained using an Ultramicrotome (Power tome PC, RMC Boeckeler^®^) with a diamond knife. Sections placed on copper grids were double-stained with uranyl acetate and lead citrate, according to the method described by Reynolds [25]. The grids were examined in a Hitachi H-7650 transmission electron microscope operated at an accelerating voltage of 80 kV, in the “Service d’Étude et de Recherche en Microscopie Électronique” of the University of Corsica (Corte, France).

### Cytochemistry

Sections placed on gold grids were stained according to the method developed by Thiéry [27] to detect the presence of glycogen. To do this, they were treated with periodic acid (PA), thiocarbohydrazide (TCH) and silver proteinate (SP) as follow: 30 min in 10% PA, rinsed in Milli-Q water, 2 h in TCH, rinsed in acetic solutions and Milli-Q water, 30 min in 1% SP in the dark and rinsed in Milli-Q water. Gold grids were examined as above.

## Results

**Figure 1.**
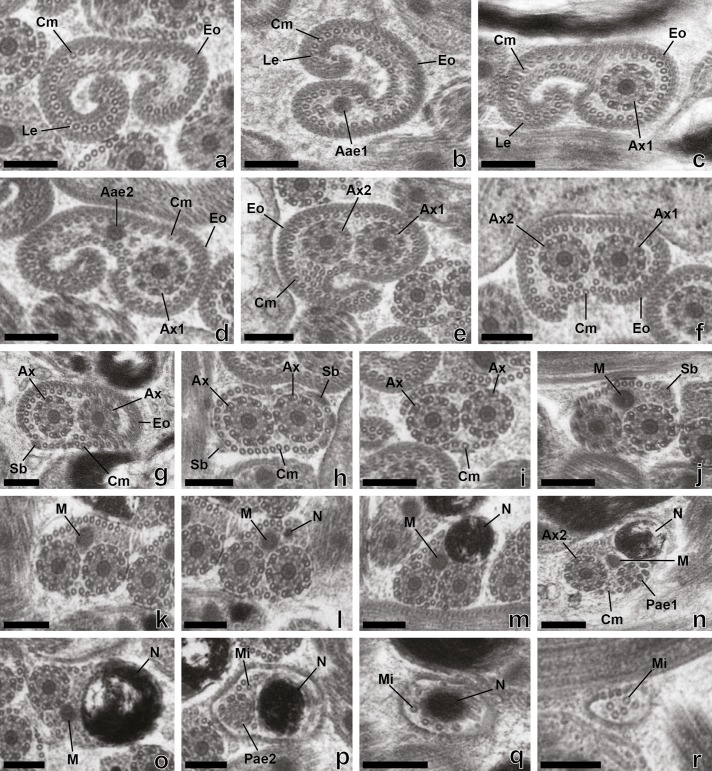
Mature spermatozoon of *Prosorhynchus longisaccatus.* (a)–(g) Consecutive cross-sections in region I of the spermatozoon showing external ornamentation of the plasma membrane, cortical microtubules under the plasma membrane, lateral expansion and appearance of both axonemes and spine-like bodies. (h)–(n) Cross-sections in region II of the spermatozoon characterized by the disappearance of the external ornamentation, presence of spine-like bodies, appearance of the mitochondrion and the nucleus, a progressive decrease of the submembranous cortical microtubules and the disorganization of the first axoneme. (o)–(r). Cross-sections in the posterior region of the mature spermatozoon with only one axoneme associated with the mitochondrion, nucleus and some cortical microtubules under the plasma membrane. This region is characterized by the progressive disappearance of the mitochondrion, the second axoneme and then the nucleus. The posterior extremity of this region exhibits only singlets resulting from the disorganization of the second axoneme. Aae1 = anterior extremity of the first axoneme, Aae2 = anterior extremity of the second axoneme, Ax1 = first axoneme, Ax2 = second axoneme, Ax = axoneme, Cm = cortical microtubule, Eo = external ornamentation, Le = lateral expansion, M = mitochondrion, Mi = microtubule, N = Nucleus, Pae1 = posterior extremity of the first axoneme, Pae2 = posterior extremity of the second axoneme, Sb = spine-like body. Scale Bars = 0.2 μm.

**Figure 2.**
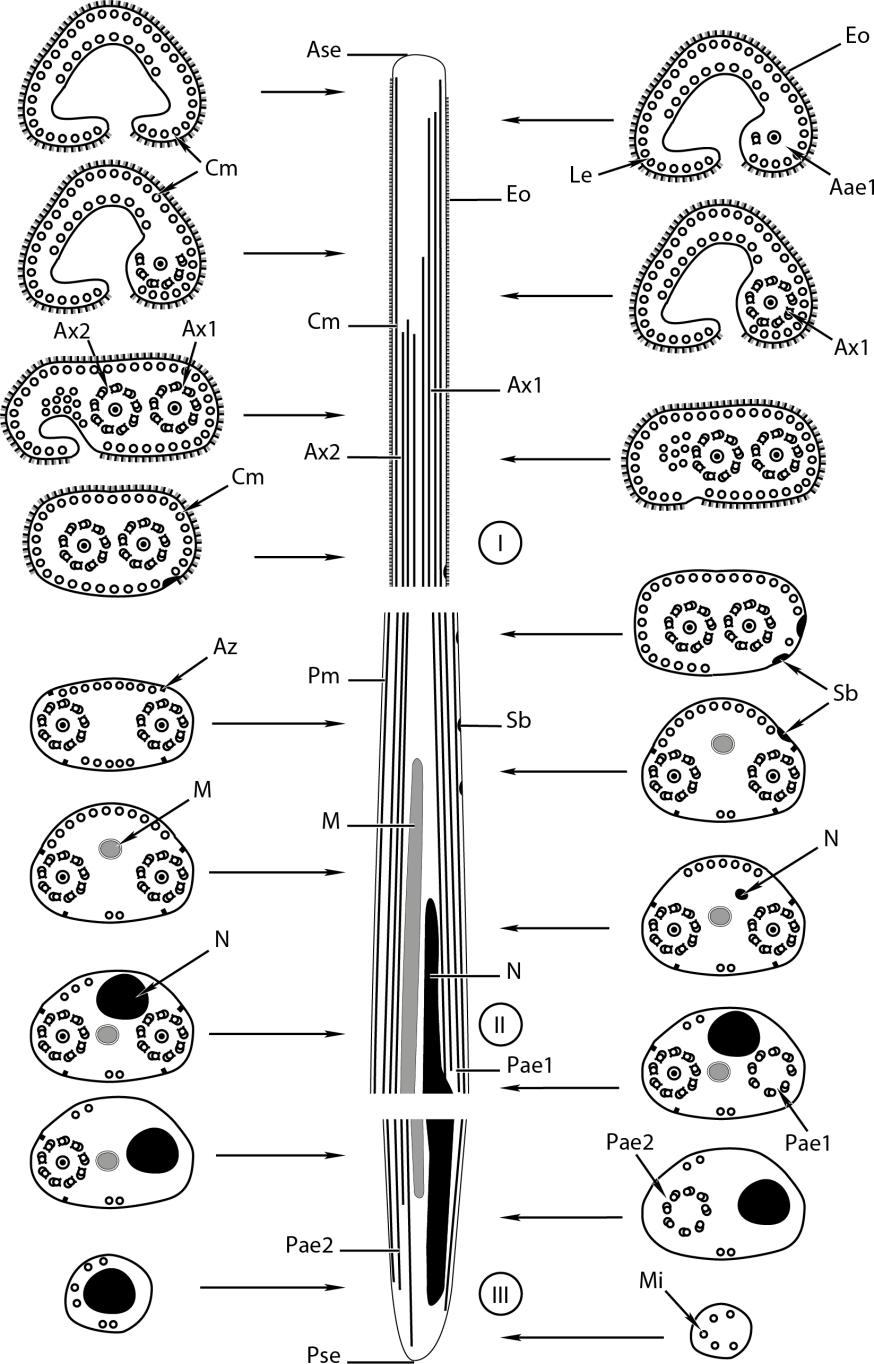
A schematic reconstruction of the mature spermatozoon of *Prosorhynchus longisaccatus.* Aae1 = anterior extremity of the first axoneme, Ase = anterior spermatozoon extremity, Ax1 = first axoneme, Ax2 = second axoneme, Az = attachment zones, Cm = cortical microtubule, Eo = external ornamentation, Le = lateral expansion, M = mitochondrion, Mi = microtubule, N = nucleus, Pm = plasma membrane, Pae1 = posterior extremity of the first axoneme, Pae2 = posterior extremity of the second axoneme, Pse = posterior spermatozoon extremity, Sb = spine-like body.

**Figure 3.**
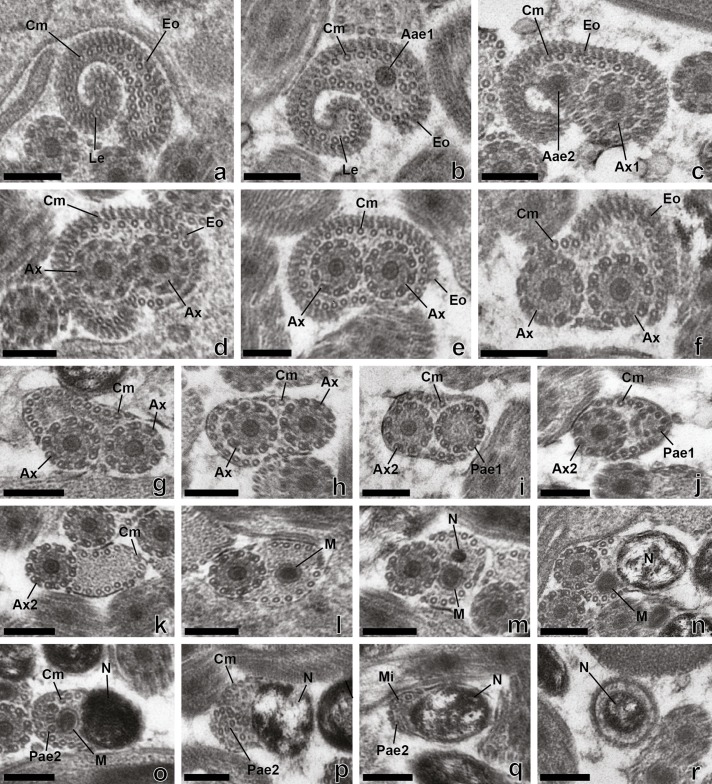
Mature spermatozoon of *Rhipidocotyle khalili.* (a)–(f) Cross-sections in the anterior region of the spermatozoon showing the external ornamentation of the plasma membrane, a submembranous layer of cortical microtubules, the lateral expansion and a progressive appearance of the two axonemes. (g)–(m) Consecutives cross-sections in region II of the spermatozoon with only two axonemes and cortical microtubules (g)–(h) More posteriorly, disorganization and disappearance of the first axoneme (i)–(k) the progressive reduction of the number of cortical microtubules and the appearance of the mitochondrion (l) then the nucleus (M). (n)**–(**r**)** Consecutive cross-sections in the posterior region of the spermatozoon showing one axoneme, mitochondrion, nucleus and cortical microtubules (n); posterior extremity of the second axoneme, mitochondrion and nucleus (o); disappearance of the mitochondrion then of the axoneme 2 (p)–(q) Cross-section in the posterior extremity of the spermatozoon, showing only the nucleus (r). Aae1 = anterior extremity of the first axoneme, Aae2 = anterior extremity of the second axoneme, Ax1 = first axoneme, Ax2 = second axoneme, Ax = axoneme, Cm = cortical microtubules, Eo = external ornamentation, Le = lateral expansion, M = mitochondrion, Mi = microtubule, N = Nucleus, Pae1 = posterior extremity of the first axoneme, Pae2 = posterior extremity of the second axoneme. Scale Bars = 0.2 μm.

**Figure 4.**
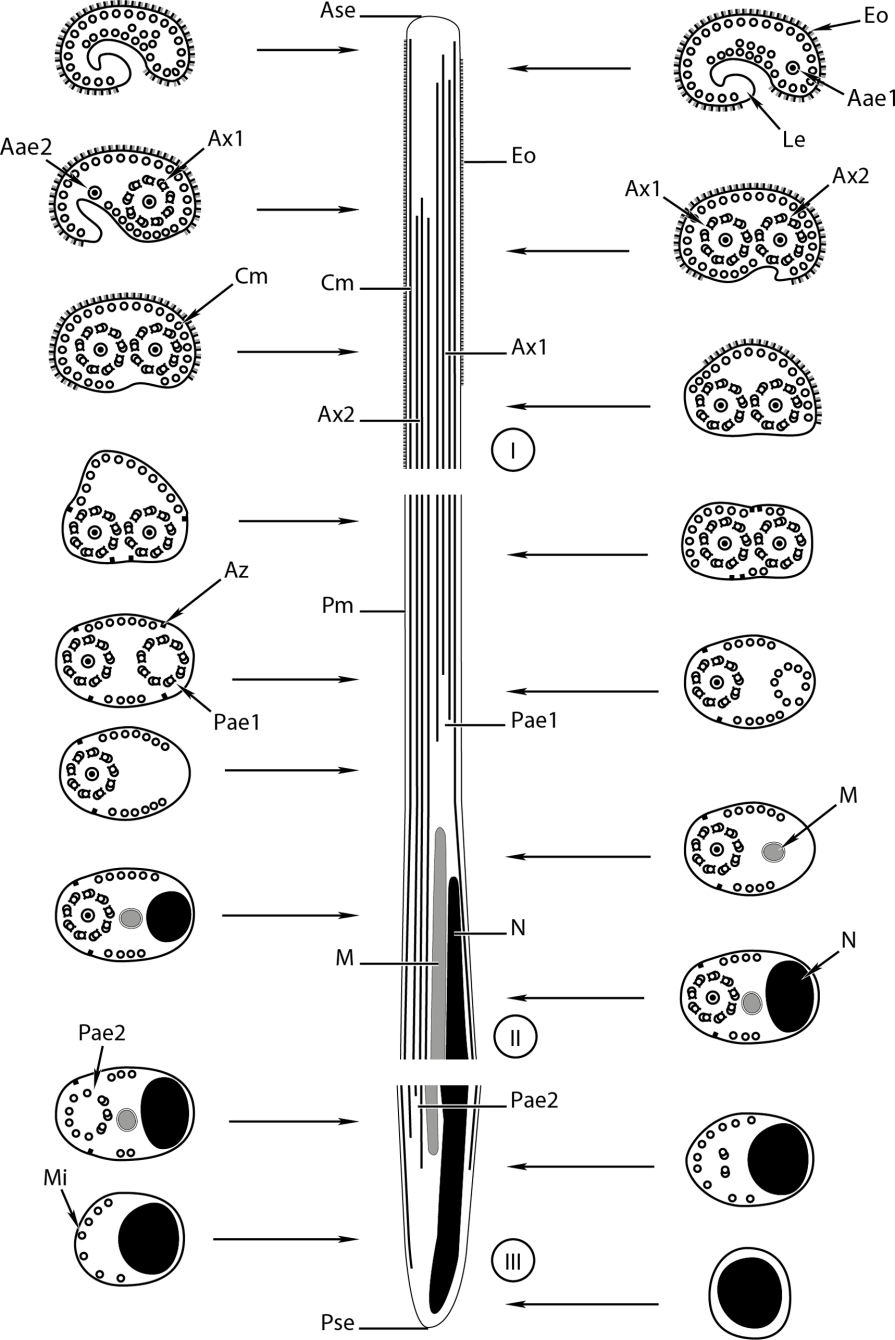
A schematic reconstruction of the mature spermatozoon of *Rhipidocotyle khalili.* Aae1 = anterior extremity of the first axoneme, Aae2 = anterior extremity of the second axoneme, Ase = anterior spermatozoon extremity, Ax1 = first axoneme, Ax2 = second axoneme, Az = attachment zones, Cm = cortical microtubule, Eo = external ornamentation, Le = lateral expansion, M = mitochondrion, Mi = microtubule, N = nucleus, Pm = plasma membrane, Pae1 = posterior extremity of the first axoneme, Pae2 = posterior extremity of the second axoneme, Pse = posterior spermatozoon extremity.

**Figure 5.**
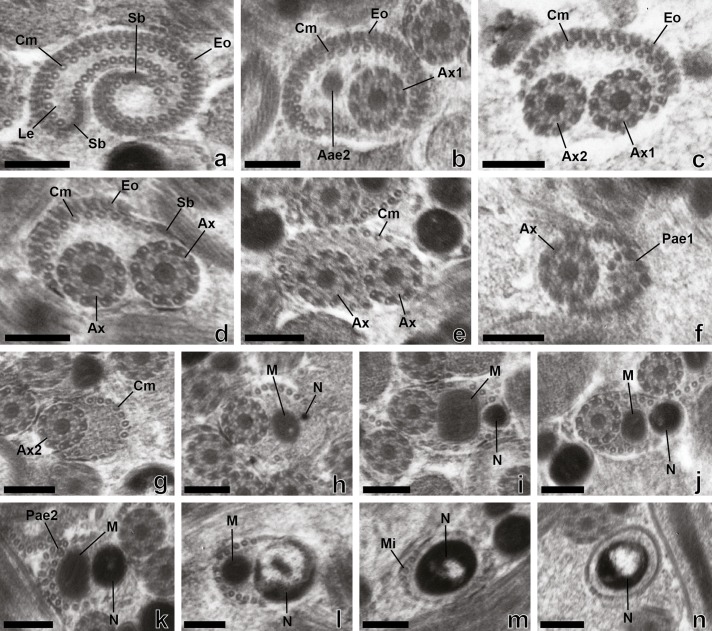
Mature spermatozoon of *Bucephalus margaritae.* (a)–(d) Cross-sections in region I of the spermatozoon showing the anterior extremity of the spermatozoon with only an external ornamentation of the plasma membrane, cortical microtubules under the plasma membrane and the lateral expansion (a). Then, the two axonemes appear progressively (b)–(c). In addition to the previous structures, the posterior extremity of this region exhibits undeveloped spine-like bodies (d). (e)–(i) Cross-section in region II of the mature spermatozoon showing only the two axonemes and cortical microtubules (e); disorganization of axoneme 1 (f); only one axoneme and cortical microtubules (g); appearance of the mitochondrion and the nucleus (h–i). (j)–(n) Cross-sections in the posterior region of the mature spermatozoon showing only the second axoneme, mitochondrion and nucleus (j), then disorganization of the axoneme (k), disappearance of the second axoneme (l), disappearance of the mitochondrion, and finally the posterior extremity of the mature spermatozoon with only the nucleus (m)–(n). Aae2 = anterior extremity of the second axoneme, Ax1 = first axoneme, Ax2 = second axoneme, Ax = axoneme, Cm = cortical microtubules, Eo = external ornamentation, Le = lateral expansion, M = mitochondrion, Mi = microtubule, N = Nucleus, Pae1 = posterior extremity of the first axoneme, Pae2 = posterior extremity of the second axoneme, Sb = spine-like body. Scale Bars = 0.2 μm.

**Figure 6.**
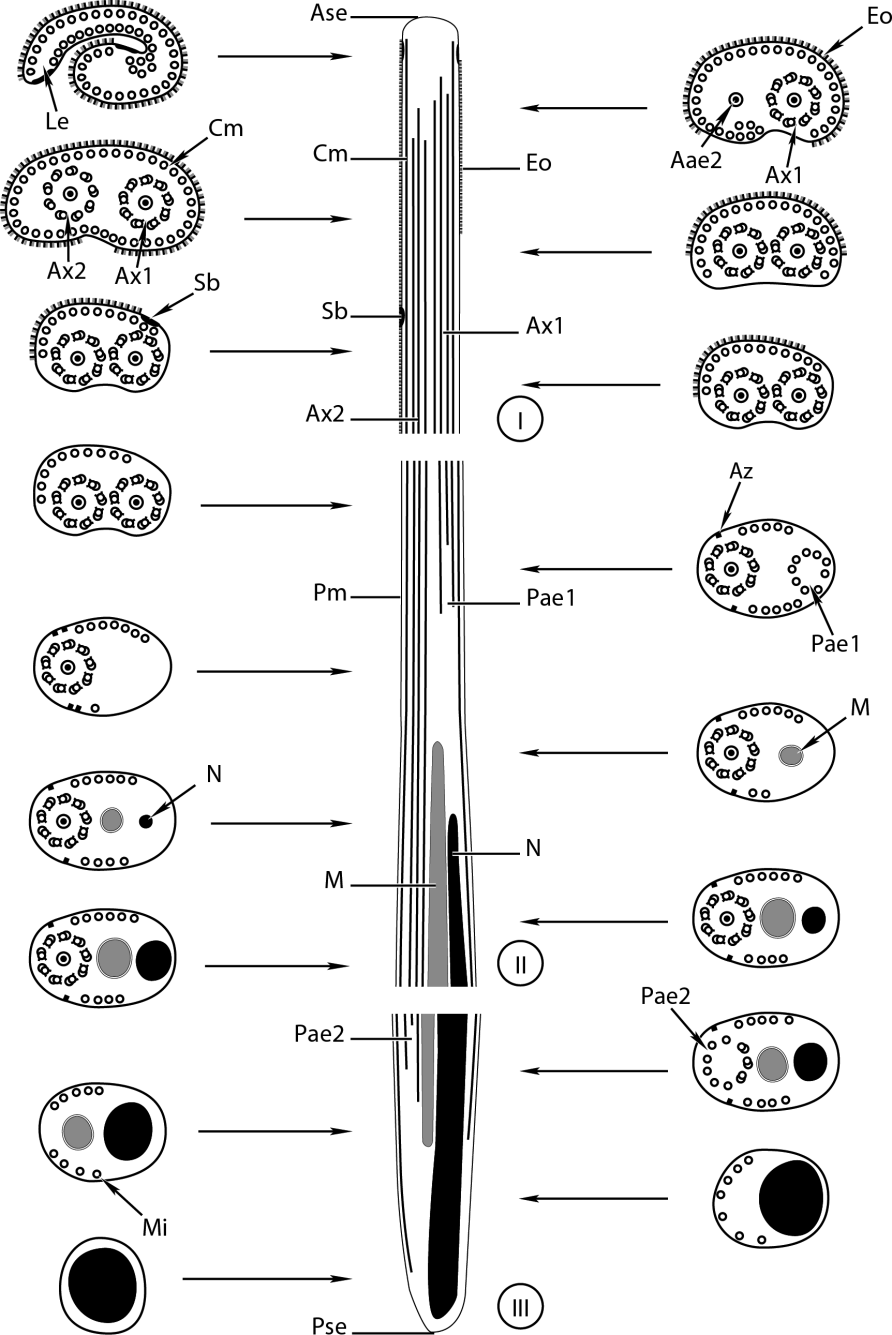
A schematic reconstruction of the mature spermatozoon of *Bucephalus margaritae.* Aae2 = anterior extremity of the second axoneme, Ase = anterior spermatozoon extremity, Ax1 = first axoneme, Ax2 = second axoneme, Az = attachment zones, Cm = cortical microtubule, Eo = external ornamentation, Le = lateral expansion, M = mitochondrion, Mi = microtubule, N = nucleus, Pm = plasma membrane, Pae1 = posterior extremity of the first axoneme, Pae2 = posterior extremity of the second axoneme, Pse = posterior spermatozoon extremity, Sb = spine-like body.

Observations of numerous longitudinal and transverse sections of the mature spermatozoon of *Prosorhynchus longisaccatus* (Figs. 1 and 2), *Rhipidocotyle khalili* (Figs. 3 and 4) and *Bucephalus margaritae* (Figs. 5 and 6) at different levels of the seminal vesicles of these worms have enabled us to distinguish three regions (I–III) in their spermatozoa from the anterior to posterior extremities (Figs. 2, 4 and 6).

Region I (Figs. 1a–1g, 2I, 3a–3f, 4I, 5a–5d, 6I) corresponds to the anterior extremity of the mature spermatozoon. The anterior extremity of this region is characterized by the presence of an external ornamentation of the plasma membrane, a lateral expansion of the plasma membrane and cortical microtubules under the plasma membrane, respectively in *P. longisaccatus* (Fig. 1a), *R. khalili* (Fig. 3a), and *B. margaritae* (Fig. 5a). Then, the first and the second axoneme appear progressively (Figs. 1b–1d, 3b–3c, 5b). The posterior extremity of this region is characterized by the presence of two axonemes, external ornamentation of the plasma membrane and cortical microtubules under the plasma membrane (Figs. 1e–1f, 3d–3e, 5c). Then, the external ornamentation disappears progressively (Figs. 1g, 3f, 5c). In these three bucephalids, the maximum number of cortical microtubules is observed in the anterior extremities of the spermatozoon, respectively 52 in *B. margaritae* (Figs. 5a, 5d), 48 in *P. longisaccatus* (Fig. 1a), and 38 in *R. khalili* (Fig. 3a). The presence of undeveloped spine-like bodies was also observed along the anterior extremity of the spermatozoon of *B. margaritae* (Fig. 5a), in the posterior extremity of region I and in the anterior part of region II of the spermatozoon of *P. longisaccatus* (Figs. 1g, 1h, 1j). The number of cortical microtubules decreases toward the posterior extremities of the spermatozoon.

Region II (Figs. 1h–1n, 2II, 3g–3m, 4II, 5e–5i, 6II) corresponds to the middle part of the spermatozoon. There is no external ornamentation of the plasma membrane and the number of cortical microtubules under the plasma membrane is reduced in all three species. In *P. longisaccatus,* the mature spermatozoon also exhibits the undeveloped spine-like bodies in this region (Figs. 1h, 1j), the appearance of the mitochondrion (Figs. 1j–1n), the nucleus (Figs. 1l–1n), and the disappearance of the first axoneme (Fig. 1n). The posterior extremity of this region exhibits only one axoneme, the disorganization of the axoneme 1, a reduced number of cortical microtubules (3), the mitochondrion, and the nucleus (Fig. 1n). In *R. khalili,* one can see the presence of only the two axonemes and cortical microtubules in the anterior part (Figs. 3g–3h) and then the disappearance of the first axoneme (Figs. 3i–3k), and the appearance of the mitochondrion (Fig. 3l) and nucleus (Fig. 3m). Thus, the posterior extremity exhibits only one axoneme, a reduced number of cortical microtubules, mitochondrion and nucleus (Fig. 3m). In *B. margaritae,* we also observed the presence of the two axonemes and cortical microtubules in the anterior part (Fig. 5e), the reduction of the number of cortical microtubules towards this region, the disorganization and disappearance of the first axoneme (Figs. 5f, 5g), and the appearance of the mitochondrion and the nucleus (Figs. 5h, 5i). The posterior extremity of this region contains only one axoneme, mitochondrion, nucleus and six cortical microtubules (Fig. 5i).

Region III (Figs. 1o–1r, 2III, 3n–3r, 4III, 5j–5n, 6III) contains, in its anterior part, one axoneme, the mitochondrion, the nucleus and similar numbers of cortical microtubules (4, 7, 8) in *P. longisaccatus, R. khalili* and *B. margaritae,* respectively (Figs. 1o, 3n, 5j). Then, one can see the disappearance of the mitochondrion, the second axoneme and the nucleus toward the posterior extremity of the spermatozoon of *P. longisaccatus* (Figs. 1p–1r). In this species, the posterior extremity of the mature spermatozoon exhibits only microtubules (Fig. 1r). In *R. khalili,* one can also see the disappearance of the mitochondrion, then the disorganization of the second axoneme (Figs. 3o–3q). The posterior extremity of the spermatozoon exhibits only the nucleus (Fig. 3r). In *B. margaritae* the posterior part of the spermatozoon is characterized by the disorganization of axoneme 2 and the disappearance of the mitochondrion (Figs. 5k–5m). The posterior extremity of the spermatozoon exhibits only the nucleus (Fig. 5n).

**Figure 7.**
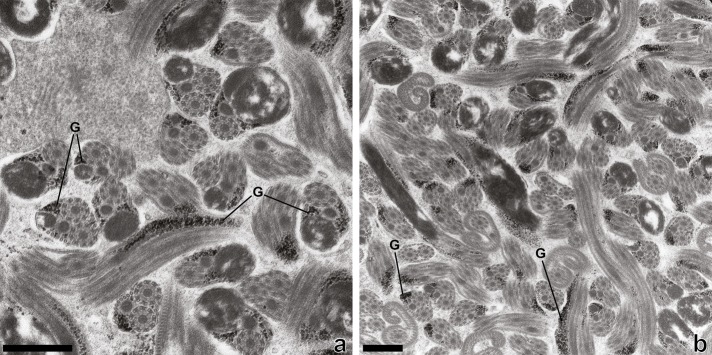
Transmission electron micrographs of spermatozoa of *Prosorhynchus longisaccatus* showing glycogen granules (G) revealed by the test of Thiéry. Scale Bars = 500 nm.

The glycogen granules are clearly highlighted with the Thiéry method, and micrographs (Figs. 7a, 7b) show their presence in regions II and III of the mature spermatozoon of *Prosorhynchus longisaccatus.*

## Discussion

Mature spermatozoa of *Prosorhynchus longisaccatus, Rhipidocotyle khalili* and *Bucephalus margaritae* exhibit the general pattern of the spermatozoon as described in most of the digeneans [4, 6, 7, 15–18, 22–24]. However, new ultrastructural data provided in this study allowed us to have an overview of the pattern in the Bucephalinae and Prosorhynchinae. In the Bucephalinae, the descriptions of the mature spermatozoa of *R. khalili* and *B. margaritae* (this study) and *Prosorhynchoides arcuatus* [10] show that the sperm cell structure is homogeneous in this subfamily. Indeed, we agree that the mature spermatozoon of the Bucephalinae contains two axonemes (including one which is shorter), external ornamentation placed in the anterior part of the spermatozoon, numerous cortical microtubules decreasing in number from the anterior to posterior extremity of the spermatozoon, one mitochondrion and a nucleus in the posterior part of the spermatozoon, which exhibits a posterior extremity with only the nucleus. The presence of spine-like bodies associated with the external ornamentation in the anterior part of the spermatozoon was described only in *Prosorhynchoides arcuatus* [10] and in *B. margaritae* (this study). The type of spine-like bodies observed in the TEM micrographs of *B. margaritae* seems to be less developed than in most digeneans [13, 17], and their absence in *R. khalili* (this study) and *P. gracilescens* [5] could be interpreted as a plesiomorphy within the Bucephalidae. Nevertheless, based on the variability of the number and the frequency of these elements from one species to another [22, 24], we believe that further studies are needed to elucidate the status of this character in the mature spermatozoa of the Bucephalinae. We described in *R. khalili* and *B. margaritae* an anterior extremity of the mature spermatozoon with only external membrane ornamentation and a row constituted by a high number of cortical microtubules under the plasma membrane. TEM micrographs of *P. gracilescens* [5] show the same structures. However, Kacem and Miquel [10] also described in *Prosorhynchoides arcuatus* the presence of one axoneme in the anterior extremity of the spermatozoon. The presence of a lateral expansion of the plasma membrane in the anterior extremity of the mature spermatozoon described in *Prosorhynchoides arcuatus* by Kacem and Miquel [10] is also confirmed in *R. khalili* and *B. margaritae* (this study). In *P. gracilescens,* Erwin and Halton [5] described an anterior extremity of the mature spermatozoon with a single axoneme, mitochondrion and nucleus. However, we believe that these authors were mistaken when they described the presence of a nucleus and mitochondrion in the anterior extremity of the mature spermatozoon.

Concerning the Prosorhynchinae, *Prosorhynchoides aculeatus* [14] and *Prosorhynchus longisaccatus* (this study) present the same type of anterior extremity of the spermatozoon as the Bucephalinae. A unique difference is described in *P. longisaccatus* with the presence of spine-like bodies only in the posterior part of its anterior extremity and in the median part of the spermatozoon. Until now, the presence of spine-like bodies in the median part of the spermatozoon in Bucephalidae was described only in *P. longisaccatus* and *Prosorhynchoides arcuatus.*

The middle region of the mature spermatozoon of the studied Prosorhynchinae presents two axonemes. However, in the Bucephalinae it exhibits one axoneme and the disorganization of the first axoneme in its anterior part (this study). In *Prosorhynchoides arcuatus,* this middle part of the spermatozoon exhibits only one axoneme. Such a short axoneme in digeneans was described for the first time in Bucephalinae by Kacem and Miquel [10] in *Prosorhynchoides arcuatus,* then in two other Bucephalinae: *B. margaritae* and *R. khalili* (this study).

The posterior extremity of the mature spermatozoon of the Bucephalidae exhibits a nucleus in Bucephalinae ([10], this study) or microtubules in Prosorhynchinae ([14], this study). These microtubules could be cortical microtubules or singlets from the second axoneme.

Mature spermatozoa of the Bucephalidae present numerous similarities with the type V described by Bakhoum et al. [1]. This typology enables these authors to classify digeneans in five types of spermatozoa according to the state of eight principal ultrastructural characters. Type V is characterized by: (1) two axonemes with the 9 + “1” pattern of the Trepaxonemata, (2) lateral expansion, (3) presence of external ornamentation, (4) association “external ornamentation + cortical microtubules”, (5) external ornamentation located in the anterior part of the spermatozoon, (6) two bundles of parallel cortical microtubules, (7) maximum number of cortical microtubules located in the anterior part of the spermatozoon, and (8) one mitochondrion in general. Table 1 shows the homogeneity of the sperm structure of the Bucephalidae.

**Table 1.** Available data on the ultrastructure of the spermatozoon in the Bucephalidae.

	Spermatozoon characters													
	Principal characters								Secondary characters					
Subfamilies and species	TAx	Le	Eo	Eo + Cm	LEo	BCm	LMCm	M	Adm	Sb	Cob	Psc	Spermatozoon type	References
Bucephalinae														
*Bucephalus margaritae*	9 + “1”	+	+	+	AntA	2	AntS	1	−	+	−	N	V	Present study
*Prosorhynchoides arcuatus*	9 + “1”	+	+	+	AntA	2	AntS	1	−	+	−	N	V	[10]
*Rhipidocotyle khalili*	9 + “1”	+	+	+	AntA	2	AntS	1	−	–	−	N	V	Present study
Prosorhynchinae														
*Prosorhynchus aculeatus*	9 + “1”	+	+	+	AntA	2	AntS	1	−	+	−	Mi	V	[14]
*Prosorhynchus longisaccatus*	9 + “1”	+	+	+	AntA	2	AntS	1	−	+	−	Mi	V	Present Study

*Notes*. Adm = anterior electron-dense material, AntA = anterior part of the anterior region, AntS = anterior region of the spermatozoon, BCm = number of bundles of cortical microtubules, Cm = cortical microtubules, Cob = cytoplasmic ornamented buttons, Eo = external ornamentation of plasma membrane, Eo + Cm = association of external ornamentation with cortical microtubules, Le = lateral expansion, LEo = location of external ornamentation, LMCm = location of maximum number of cortical microtubules, M = number of mitochondria, Mi = microtubule, N = nucleus, Psc = posterior spermatozoon character, Sb = spine-like bodies, TAx = type of axoneme, +/− = presence/absence of considered character.

## Conclusion

Our study increases the ultrastructural data on the mature spermatozoon in the Bucephalidae, and provides characters that might be useful for phylogenetic purposes. However, the lack of ultrastructural data on the mature spermatozoon in the other three recognized subfamilies of Bucephalidae (Dolichoenterinae, Heterobucephalopsinae and Paurorhynchinae) justifies the need for more ultrastructural studies to define a spermatozoon model for the Bucephaloidea superfamily. Nevertheless, we conclude that mature spermatozoa of *Prosorhynchus longisaccatus, Rhipidocotyle khalili* and *Bucephalus margaritae* exhibit all the structures described to date in the Bucephalidae, namely (i) two axonemes of the 9 + “1” pattern of Trepaxonemata with different lengths; the difference in length is variable according to the genus; (ii) the anterior extremity of the mature spermatozoon exhibits only the anterior extremity of the axoneme 1, a lateral expansion and extramembranar ornamentation of the plasma membrane and a complete row of cortical microtubules under the plasma membrane; (iii) the mitochondrion and nucleus are located in the posterior part of the spermatozoon; and (iv) there is variation in the type of posterior extremity of mature spermatozoa according to the subfamily.

## Conflict of interest

The Editor-in-Chief of Parasite is one of the authors of this manuscript. COPE (Committee on Publication Ethics, https://publicationethics.org/), to which Parasite adheres, advises special treatment in these cases. In this case, the peer-review process was handled by an Invited Editor, Jérôme Depaquit.
